# Hybrid surgery of percutaneous transforaminal endoscopic surgery (PTES) combined with OLIF and anterolateral screws rod fixation for treatment of multi-level lumbar degenerative diseases with intervertebral instability

**DOI:** 10.1186/s13018-023-03573-3

**Published:** 2023-02-17

**Authors:** Tianyao Zhou, Yutong Gu

**Affiliations:** 1 Department of Orthopaedic Surgery, Zhongshan Hospital Fudan University, Shanghai, 200032 China; 2 Shanghai Southwest Spine Surgery Center, Shanghai, 200032 China

**Keywords:** Lumbar degenerative disease, multi-level, intervertebral instability, Minimally invasive, Spine surgery, Percutaneous transforaminal endoscopic, Oblique, Interbody fusion, Screws rod fixation

## Abstract

**Background:**

Oblique lumbar interbody fusion (OLIF) has been used to treat lumbar intervertebral instability, which has some advantages including less trauma, less blood loss, faster recovery and bigger cage. However, it usually needs posterior screws fixation for biomechanical stability, and possible direct decompression for relieving neurologic symptoms. In this study, OLIF and anterolateral screws rod fixation through mini-incision were combined with percutaneous transforaminal endoscopic surgery (PTES) for the treatment of multi-level lumbar degenerative diseases (LDDs) with intervertebral instability. The purpose of study is to evaluate the feasibility, efficacy and safety of this hybrid surgery.

**Methods:**

From July 2017 to May 2018, 38 cases of multi-level LDDs of disc herniation, foramen stenosis, lateral recess stenosis or central canal stenosis with intervertebral instability and neurologic symptoms undergoing one-stage PTES combined with OLIF and anterolateral screws rod fixation through mini-incision were recruited in this retrospective study. The culprit segment was predicted according to the position of patient’s leg pain and PTES under local anesthesia was performed for the culprit segment in the prone position to enlarge the foramen, remove the flavum ligamentum and herniated disc for the lateral recess decompression and expose bilateral traversing nerve roots for the central spinal canal decompression through an unilateral incision. During the operation, communicate with the patients to confirm the efficacy using VAS. And then mini-incision OLIF using allograft, autograft bone harvested in PTES and anterolateral screws rod fixation were performed in the right lateral decubitus position under general anesthesia. Back and leg pain were preoperatively and postoperatively evaluated using VAS. And the clinical outcomes were evaluated with ODI at the 2-year follow-up. The fusion status was assessed according to Bridwell’s fusion grades.

**Results:**

There were 27 cases of 2-level, 9 cases of 3-level and 2 cases of 4-level LDDs with single-level instability on the X-ray, CT and MRI. Five cases of L3/4 instability and 33 cases of L4/5 instability were included. PTES was performed for 1 segment of 31 cases (25 cases of instability segment, 6 cases of no instability segment) and 2 segments including instability segment of 7 cases. Then, all instability segments were treated using mini-incision OLIF and anterolateral screws rod fixation. The average operation duration was 48.9 ± 7.3 min per level for PTES and 69.2 ± 11.6 min for OLIF and anterolateral screws rod fixation. The mean frequency of intraoperative fluoroscopy was 6 (5–9) times per level for PTES and 7 (5–10) times for OLIF. There was a mean blood loss of 30 (15–60) ml, and the incision length was 8.1 ± 1.1 mm for PTES and 40.0 ± 3.2 mm for OLIF. The mean hospital stay was 4 (3–6) days. The average follow-up duration was 31.1 ± 4.0 months. For the clinical evaluation, the VAS pain index and the ODI showed excellent outcomes. Fusion grades based on the Bridwell grading system at 2-year follow-up were grade I in 29 segments (76.3%) and grade II in 9 segments (23.7%). One patient encountered nerve root sleeves rupture during PTES and did not confront cerebrospinal fluid leakage or other abnormal clinical symptoms. There were two cases of hip flexion pain and weakness, which was relieved during 1 week after surgery. No patients had any form of permanent iatrogenic nerve damage and a major complication. No failure of instruments was observed.

**Conclusions:**

The hybrid surgery of PTES combined with OLIF and anterolateral screws rod fixation is a good choice of minimally invasive surgery for multi-level LDDs with intervertebral instability, which can get direct neurologic decompression, easy reduction, rigid fixation and solid fusion, and hardly destroy the paraspinal muscles and bone structures.

**Supplementary Information:**

The online version contains supplementary material available at 10.1186/s13018-023-03573-3.

## Introduction

Lumbar degeneration and spondylolysis are the main reasons for lumbar intervertebral instability to happen [1, 2]. When conservative treatment fails, lumbar interbody fusion and neurologic decompression become the standard surgical treatment. Lumbar interbody fusion surgery was initially invented to treat spinal tuberculosis [3, 4]. In 1948, Lane and Moore [5] first applied lumbar interbody fusion for the treatment of lumbar degenerative diseases and obtained encouraging result of relieving symptoms. Since then, the indication of lumbar interbody fusion has been widened. Nowadays, lumbar interbody fusion is applied to patients with lumbar disc herniation, spondylolisthesis, pseudoarthrosis and spinal deformities [6].

In 1997, Mayer [7] reported an anterior to psoas surgical trajectory for lumbar interbody fusion. In 2012, Silvestre et al. [8] named the approach oblique lumbar interbody fusion (OLIF). OLIF has been used to treat lumbar spine spondylolisthesis, which has some advantages including less damage to paraspinal muscles and bone structures, less blood loss and faster recovery. Compared with posterior lumbar interbody fusion (PLIF) or transforaminal lumbar interbody fusion (TLIF), OLIF uses bigger cage to achieve higher fusion rate by getting more touch surface between endplate of vertebra and cage and implanting more graft bone [9, 10]. In addition, bigger cage has better distraction ability of intervertebral space helpful for restoration of intervertebral space height and lumbar lordotic angle, reduction of spondylolisthesis.

However, posterior instrumentation was usually needed to enhance the biomechanical stability of OLIF [11]. Sometimes there was no improvement of neurologic symptoms after surgery due to indirect and inadequate decompression of OLIF [10, 12], or neurologic compression in other degenerative segments except intervertebral instability. Further posterior surgery sharply reduced the advantages of OLIF resulting from longer operative time under general anesthesia and more invasiveness [13]. In this study, OLIF and anterolateral screws rod fixation in the same mini-incision were combined with percutaneous transforaminal endoscopic surgery (PTES) [14–16] under local anesthesia for the treatment of multi-level lumbar degenerative diseases (LDDs) with intervertebral instability in order to obtain direct decompression, good reduction, rigid fixation, high fusion rate, and protect the paraspinal muscles and bone structures as much as possible. The purpose of study was to evaluate the feasibility, efficacy and safety of this hybrid surgery.

## Materials and methods

### Patients

The clinical study proposal was approved by the medical ethics committee of Zhongshan Hospital Fudan University. Informed consent was obtained from all individual participants for using their imaging data and questionnaire scores. From July 2017 to May 2018, 38 cases of multi-level LDDs with intervertebral instability and neurologic symptoms undergoing one-stage PTES combined with OLIF and anterolateral screws rod fixation through mini-incision were recruited in this retrospective study.

**Fig. 1:**
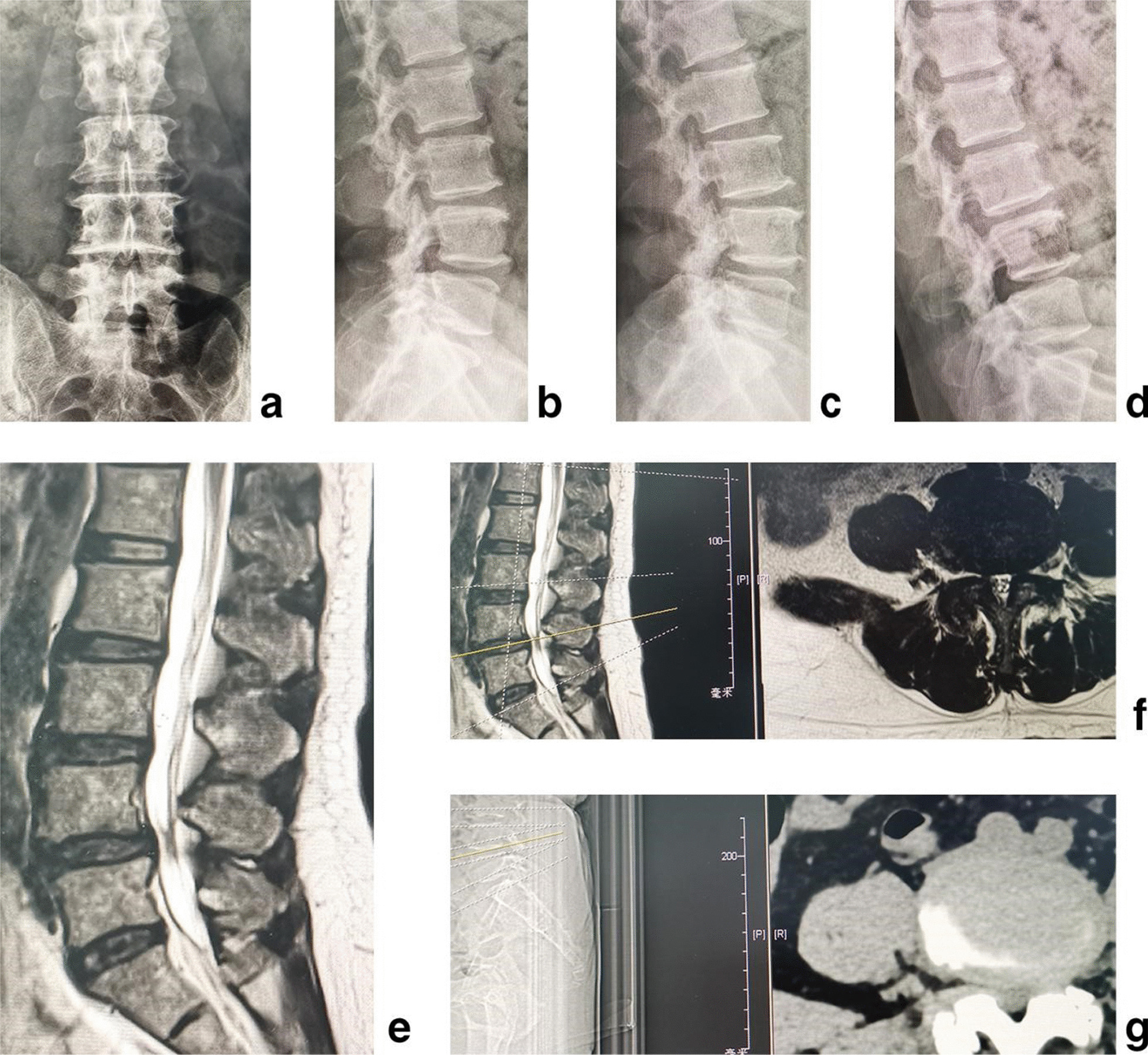
The female patient of 58 years old had the pain of right lateral buttock, posterolateral thigh, lateral calf and dorsal foot. **a** Posteroanterior X-ray, **b** lateral X-ray, **c** hyperextension lateral X-ray, **d** hyperflexion lateral X-ray, **e** sagittal MR image, **f** axial MRI and **g** axial CT showed multi-level lumbar degeneration of L3/4, L4/5, L5/S1 with L4/5 instability. It was diagnosed with multi-level LDDs and L4/5 instability

The inclusion criteria were as follows: (1) low back pain, and unilateral or bilateral leg pain, or intermittent claudication with no symptom of legs when rest and pain, numbness, discomfort or tiredness of unilateral or bilateral leg after walking 50 to 100 m, unable to walk, relieved after rest. (2) Image data of X-ray, MRI and CT showed multi-level lumbar degeneration of disc herniation, intervertebral foramen stenosis, lateral recess stenosis or central spinal canal stenosis with intervertebral instability, which was forward or backward displacement of adjacent vertebral bodies > 3 mm or change of intervertebral angle > 15° on the hyperextension and hyperflexion lateral X-ray, or lumbar spondylolisthesis (Meyerding [17] I° or II°) (Fig. 1a–g). (3) Outcome was poor after at least 3 months of regular conservative treatment, and symptoms severely affect work and daily life. (4) The systemic status was good, basic medical diseases such as heart disease, hypertension or diabetes were under control, and the mental state was normal. 5. With complete data and perioperative records, as well as radiographic follow-up data.

The exclusionary criteria were the presence of L5/S1 instability, previous lumbar interbody fusion, spinal tumor, spinal infection, other medical conditions making the patient intolerant to operation, inability to give informed consent, and a likelihood of noncompliance with follow-up.

### Pre- and postoperative imaging

All patients were evaluated before the procedure by CT and MRI imaging to determine lumbar disc herniation, lateral recess stenosis, intervertebral foramen stenosis or central spinal canal stenosis. Posteroanterior, lateral radiographs and hyperextension, hyperflexion lateral X-ray were obtained to assess intervertebral instability or the slip degree of vertebral body according to the Meyerding Classification System of Spondylolisthesis [17]. Intervertebral space height [18], lumbar lordotic angle and operative segmental lordotic angle were measured on lumbar spine lateral X-rays at preoperative, postoperative and 2-year follow-up. The intervertebral space height is the average of anterior and posterior spaces between two adjacent vertebrae on the lateral X-ray; the lumbar lordotic angle is the angle between the upper endplate of first lumbar vertebra and the upper endplate of sacrum; the operative segmental lordotic angle is the angle between the upper endplate of upper vertebra and the lower endplate of lower vertebra in the surgical segment. Postoperative X-ray and CT are used to assess the position of internal fixation. A loss of at least 2 mm of intervertebral space height is generally considered cage subsidence on X-ray [18]. The fusion status is assessed by a senior orthopedic surgeon according to the Bridwell’s fusion grades on CT [19]. After the treatment, MRI images were obtained to exclude dural cyst, myelomeningocele, dural tears or spinal fluid leaks, and reherniation.

### Surgical procedure

All the surgeries were undertaken by the same senior surgeon. C-arm was used for intraoperative fluoroscopic imaging.

The culprit segment was predicted according to the position of patient’s leg pain. The central buttock, posterior thigh, posterior calf, lateral malleolus, or plantar: S1 nerve root is involved, and the culprit segment is generally L5/S1. The lateral buttock, posterolateral thigh, lateral calf, or dorsal foot: L5 nerve root is involved, and the culprit segment is generally L4/5 (traversing nerve root) or L5/S1 (exiting nerve root). The lateral buttock, anterolateral thigh, knee, medial calf, or medial malleolus: L4 nerve root is involved, and the culprit segment is generally L3/4 (traversing nerve root) or L4/5 (exiting nerve root). The distal one-third of the anterior thigh or the medial part of the condyle: L3 nerve root is involved, and the culprit segment is generally L2/3 (traversing nerve root) or L3/4 (exiting nerve root). The middle one-third of the anterior aspect of the thigh: L2 nerve root is involved, and the culprit segment is generally L1/2 (traversing nerve root) or L2/3 (exiting nerve root). The proximal one-third of the anterior aspect of the thigh: L1 nerve root is involved, and the culprit segment is generally T12/L1 (traversing nerve root) or L1/2 (exiting nerve root). The first target is the segment involving traversing nerve root because the proportion of far lateral lumbar disc herniation or intervertebral foramen stenosis involving exiting nerve root in LDDs is relatively lower [16].

**Fig. 2:**
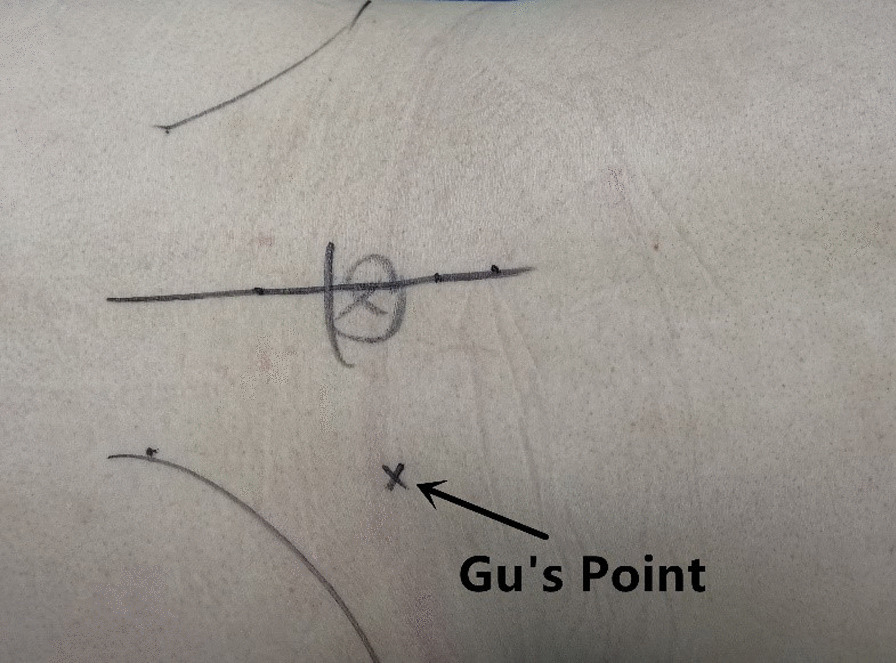
Photography shows the surface marking of anatomic disc center identified by the intersection of transverse line and longitudinal midline, which is the aiming reference point of puncture, and the entrance point of puncture (Gu’s point) locates at the corner of the flat back turning to the lateral side

**Fig. 3:**
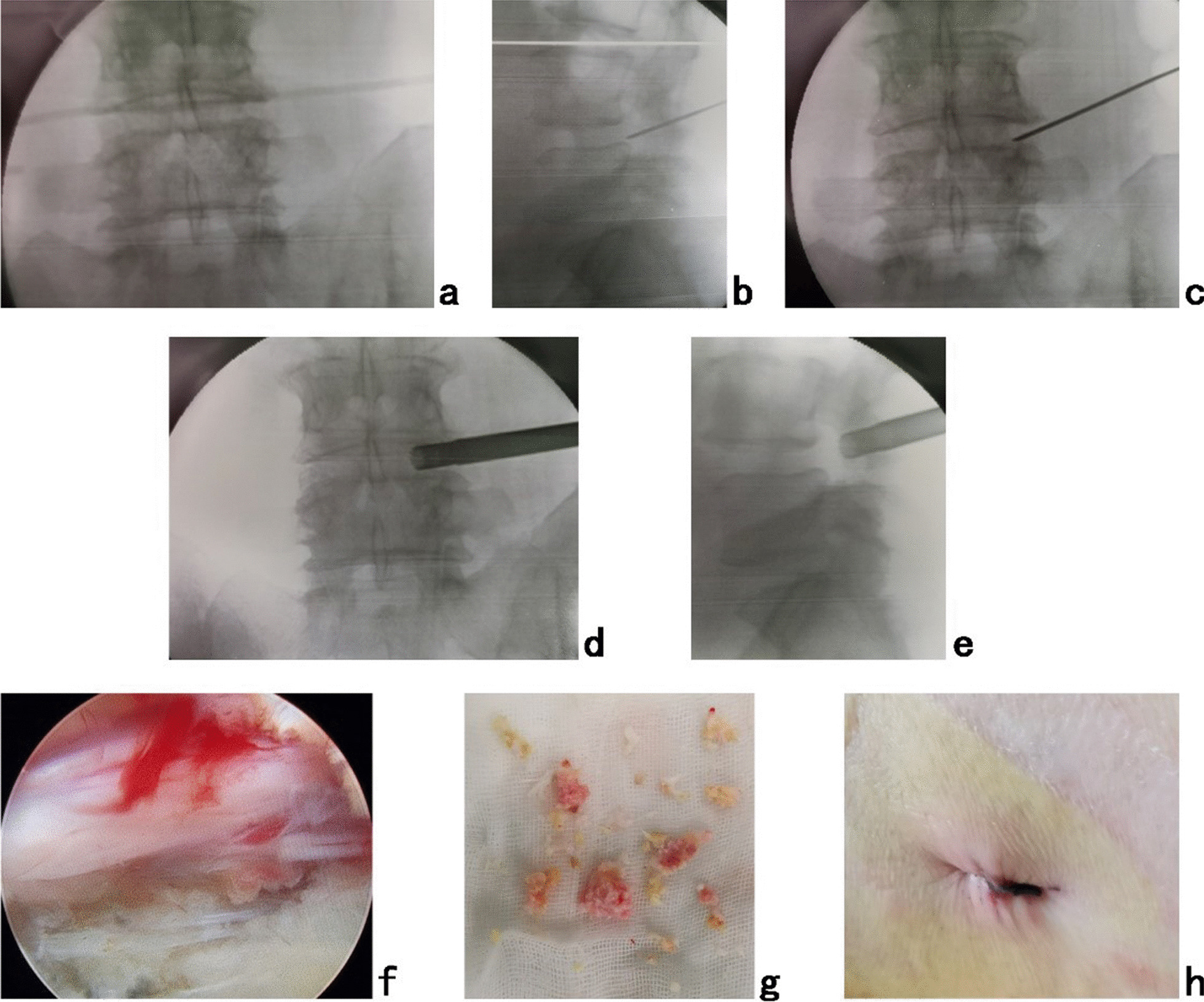
The patient had the pain of right lateral buttock, posterolateral thigh, lateral calf, and dorsal foot, which indicates L5 nerve root is involved, and the culprit segment was predicted as L4/5. PTES was performed under local anesthesia for L4/5. A transverse line bisecting L4/5 disc was drawn along the metal rod which was placed transversely across the center of the target disc on **a** posteroanterior C-arm view. After successful puncture, the C-arm view was taken to ensure that the needle reached the target. The tip of puncture needle was in the intracanal area close to the posterior wall of the disc on **b** lateral X-ray and near the lateral border of the pedicle on **c** posteroanterior X-ray. During press-down enlargement of foramen, when resistance disappeared, the tip of reamer should exceed the medial border of the pedicle on **d** posteroanterior C-arm view and reach close to the posterior wall of the target disc on **e** lateral C-arm view. Under **f** endoscopic view, the compressed nerve root was freed after **g** the hypertrophic ligmentum flavum and herniated disc were removed. **h** The stab incision of 8 mm was closed after confirming the efficacy

The patient was placed in a prone position on a radiolucent table for PTES under local anesthesia with conscious sedation. The entrance point of puncture locates at the corner of the flat back turning to the lateral side at the height of target disc, or cranially or slightly caudally (Fig. 2). This entrance point, named “Gu’s point”, is easy to determine without the fluoroscopy regardless of different age, gender and body size [14–16]. The intersection of posterior midline and the transverse line of surface marking of target disc is the aiming reference point of puncture (Figs. 2, 3a), and an 18-gauge puncture needle was inserted anteromedially at an angle of about 45°(25°-85°) to horizontal plane. After the success of puncture (Fig. 3b, c) and dilating the puncture tract stepwise, an 8.8-mm-diameter cannula with one-side opening was inserted over the guiding rod and docked at the facet joint. Then press down the cannula to decrease the inclination angle and a 7.5-mm-diameter hand reamer was introduced through the cannula to remove the ventral bone of articular process to enlarge the foramen. When resistance disappeared, the tip of reamer should exceed the medial border of pedicle on posteroanterior view and reach close to the posterior wall target disc on lateral view (Fig. 3d, e). This enlargement procedure is named “press-down enlargement of foramen” [14–16]. The 7.5-mm-diameter working cannula was inserted over the guiding rod, and under endoscope (Fig. 3f), the hypertrophic ligmentum flavum and herniated disc (Fig. 3g) were removed to expose the ipsilateral traversing and exiting nerve root for lateral recess decompression, even the contralateral traversing nerve root for central spinal canal decompression. Sometimes the central and contralateral posterior longitudinal ligament, annulus fibrosus or nucleus pulposus needed to be removed using flexible bipolar radiofrequency and angled nucleus pulposus forceps for clearer exposure of contralateral nerve root.

During the operation under local anesthesia, communication with the patients was made to confirm the efficacy. When the patient is placed in a prone position, there are generally no symptoms of leg pain and numbness, but the involved legs have an obvious sense of relaxation after neurologic decompression is achieved. We used the visual analogue scale (VAS) to evaluate the relaxation sensation of involved leg. Preoperative status of no relaxation is 10, and complete relaxation is 0: 0–3, obvious relaxation, good outcome, the treated segment is the culprit one, and the operation can be finished; 4–6, moderate relaxation, partially effective, the treated segment is the culprit one, and other culprit segments need treatment; 7–10, mild relaxation or no relaxation, no efficacy, the treated segment is not the culprit one, and the culprit segment needs treatment [16]. The two adjacent segmental operations could be completed through one stab incision of about 8 mm (Fig. 3h).

**Fig. 4:**
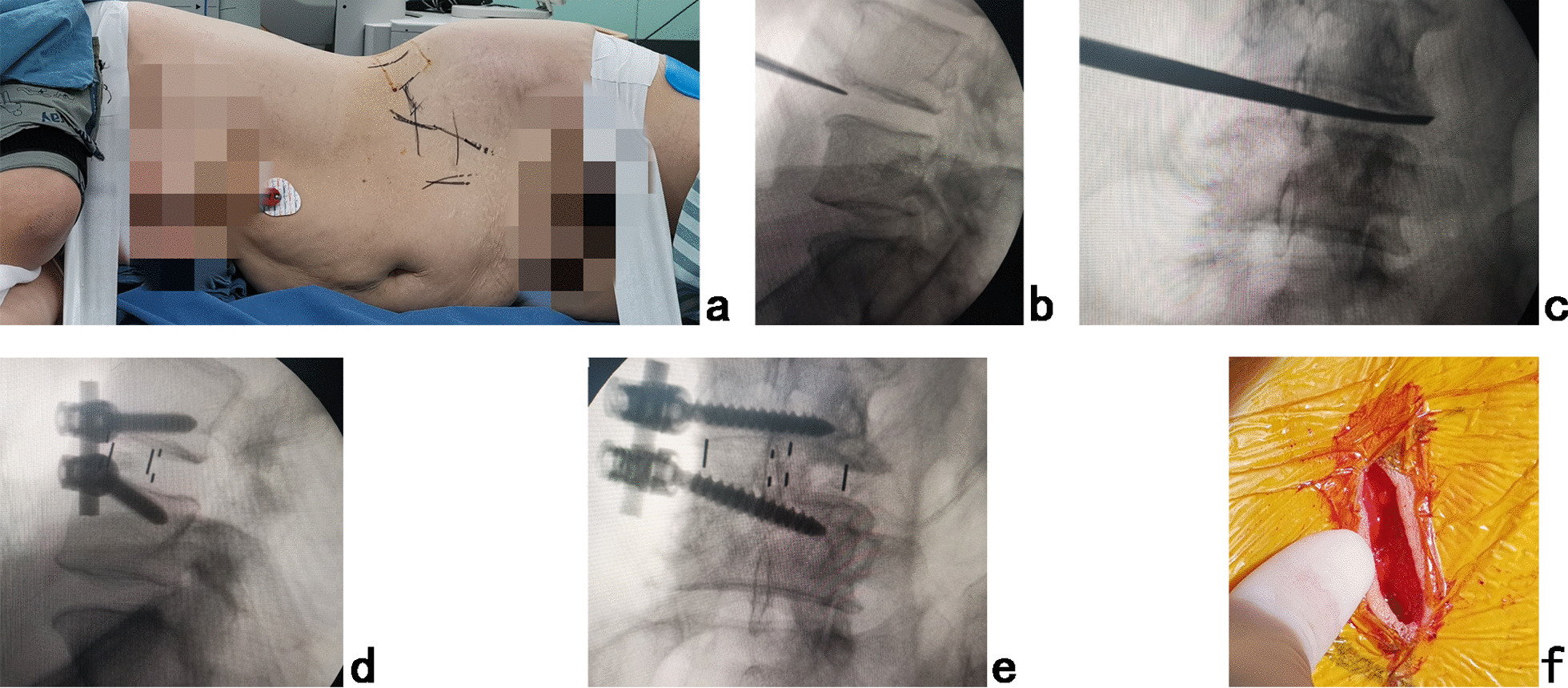
**a** Patient was placed into right lateral decubitus position for mini-incision OLIF and anterolateral screws rod fixation under general anesthesia. The target intervertebral space was positioned using **b** C-arm view. The spatula was inserted into intervertebral space to cut the contralateral fibrous annulus, which was confirmed by **c** X-ray image. After the OLIF cage was placed into disc space parallelly to the endplate and anterolateral screws rod fixation was performed, **d** lateral and **e** posteroanterior C-arm view confirmed good position of internal instruments. **f** The picture showed the mini-incision of OLIF

Then, the patients were placed into a right lateral decubitus position (Fig. 4a) under controlled general anesthesia with trachea cannula to undergo mini-incision OLIF and anterolateral screws rod fixation for instability segment. The preoperative C-arm was used to position the surface mark of anterior edge of target intervertebral space, and the mini-incision is located along the anterior edge of intervertebral space or iliac crest. After the skin and subcutaneous tissues were incised, the external oblique, internal oblique and transverse abdominal muscles were bluntly separated in turn to enter the retroperitoneal space and expose the anterior border of psoas major muscle with two narrow long retractors. After fluoroscopic projection for confirming the surgical segment (Fig. 4b), the intervertebral fibrous annulus was opened from the lateral side along the anterior border of psoas major muscle, and the spatula was inserted into intervertebral space to cut the contralateral fibrous annulus (Fig. 4c). The intervertebral tissue was removed, and upper and lower cartilage endplates were adequately scraped off, taking care to avoid damaging the bony endplates during the operation. After trial molding, the OLIF cage (Medtronic) of appropriate size was filled with allograft bone and autograft obtained during PTES, and placed into disc space parallelly to the endplate. Through the same approach, locate the entrance point of anterolateral screw at anterolateral area of vertebral body close to the adjacent endplate of OLIF cage in order to avoid the damage of segmental vessels and iliac lumbar vein, psoas. After a handheld probe was used to enter the upper vertebral body up and backward and the lower vertebral body down and backward, the trajectory integrity was verified in all 4 quadrants to be sure that a solid tube of bone existed and that violation into the spinal canal or disc had not occurred. Then, two 6.5-mm-diameter pedicle screws (Medtronic) of appropriate length were inserted into adjacent vertebrae. Finally, after the fluoroscopic view of the cage and screws was satisfying, the rod was fixed over the screws (Fig. 4d, e) and surgical incision (Fig. 4f) was closed layer by layer with a thin drain tube (Additional file 1).

Patients could walk with a flexible brace after the drain tube was removed usually 1 or 2 days after the surgery when the drainage fluid was less than 20 ml/24 h. After leaving hospital, patients were encouraged to return to daily life and followed up regularly.

### Clinical follow-up

Back and leg pain were evaluated using the 10-point VAS preoperatively, immediately, 1, 2, 3 and 6 months, 1 and 2 years after surgery. The clinical outcomes were evaluated with Oswestry Disability Index (ODI) at 2-year follow-up. During the follow-up, all complications were recorded including iatrogenic nerve damage, vascular injuries, infection, wound healing, thrombosis, or recurrence.

### Statistical analysis

All data were analyzed by SPSS version 20.0 software, and a value of less than 0.05 was considered statistical significance. The one-way ANOVA for independent samples followed by Tukey post hoc analysis was used for VAS, Intervertebral space height, lumbar lordotic angle and operative segmental lordotic angle comparisons at different time points. ODI score before the treatment and 2 years after surgery was compared using t-test.

## Results

Thirty-eight patients, 27 women and 11 men with a mean age of 63.2 ± 10.9 years, were included in the present study. There were 27 cases of 2-level, 9 cases of 3-level and 2 cases of 4-level LDDs with single-level instability on the X-ray, CT and MRI. 5 cases of L3/4 instability and 33 cases of L4/5 instability were included. PTES was performed for 1 segment of 31 cases (25 cases of instability segment, 6 cases of no-instability segment) and 2 segments including instability segment of 7 cases. Then, all instability segments were treated using mini-incision OLIF and anterolateral screws rod fixation. The patients’ characteristics are summarized in Table 1.

**Table 1:** Characteristics of the patients

Age(years)	63.2 ± 10.9	
Gender (F/M)	27/11	
Multi-level LDDs with intervertebral instability(cases)		
2-level	27	
3-level	9	
4-level	2	
Instability segment		
L3/4	5	
L4/5	33	
Segment treated with PTES(cases)		
1 segment	31	
	Instability segment	25
	No instability segment	6
2 segments (including instability)	7	
Operation duration(minutes)		
PTES	48.9 ± 7.3	
OLIF	69.2 ± 11.6	
Frequency of intraoperative fluoroscopy(times)		
PTES	6 (5–9)	
OLIF	7 (5–10)	
Blood loss(ml)	30 (15–60)	
Incision length(mm)		
PTES	8.1 ± 1.1	
OLIF	40.0 ± 3.2	
Hospital stay(days)	4 (3–6)	

The average operation duration was 48.9 ± 7.3 min per level for PTES (under local anesthesia) and 69.2 ± 11.6 min for OLIF and anterolateral screws rod fixation (under general anesthesia). The mean frequency of intraoperative fluoroscopy was 6 (5–9) times per level for PTES and 7 (5–10) times for OLIF and anterolateral screws rod fixation. There was a mean blood loss of 30 (15–60) ml, and the average incision length was 8.1 ± 1.1 mm for PTES and 40.0 ± 3.2 mm for OLIF and anterolateral screws rod fixation. The mean hospital stay was 4 (3–6) days (Table 1).

The average follow-up duration was 31.1 ± 4.0 months. The VAS of back and leg significantly dropped from 6 (4–10) and 9 (7–10) preoperatively, respectively, to 1 (0–3) (F = 313.822, *P* < 0.001) and 1 (0–4) (F = 927.809, *P* < 0.001) immediately after surgery and to 0 (0–3) (F = 511.273, *P* < 0.001) and 0 (0–2) (F = 2627.951, *P* < 0.001) at 2-year follow-up. ODI was significantly reduced from 66.8% ± 8.5% before surgery to 13.1% ± 5.8% 2 years after surgery (t = 46.068, *P* < 0.001) (Table 2).

**Table 2:** Variations of measured parameters before and after operation

	Pre-op	Post-op	1 month	2 months	3 months	6 months	1 year	2 years
Back pain VAS	6 (4–10)	1 (0–3)*	0 (0–3)*	0 (0–3)*	0 (0–3)*	0 (0–3)*	0 (0–3)*	0 (0–3)*
Leg pain VAS	9 (7–10)	1 (0–4)*	0 (0–2)*	0 (0–2)*	0 (0–2)*	0 (0–2)*	0 (0–2)*	0 (0–2)*
ODI(%)	Before operation			2 years after operation				
	66.78 ± 8.46			13.10 ± 5.81				
	Before operation		Immediately after operation				2 years after operation	
Intervertebral space height(mm)	9.77 ± 1.58		13.03 ± 1.54*				12.38 ± 1.52*	
Lumbar lordotic angle(°)	40.21 ± 9.51		48.70 ± 8.37*				46.07 ± 8.44*	
Operative segmental lordotic angle(°)	15.19 ± 3.05		20.61 ± 3.09*				19.14 ± 2.96*	

VAS is expressed as the median(minimum–maximum) and other variations are expressed as the mean ± SD. All parameters were significantly different between preoperation and postoperation (*P* < 0.001). There was no significant difference among each time point after operation

*P < 0.001 versus the parameter before surgery

**Fig. 5:**
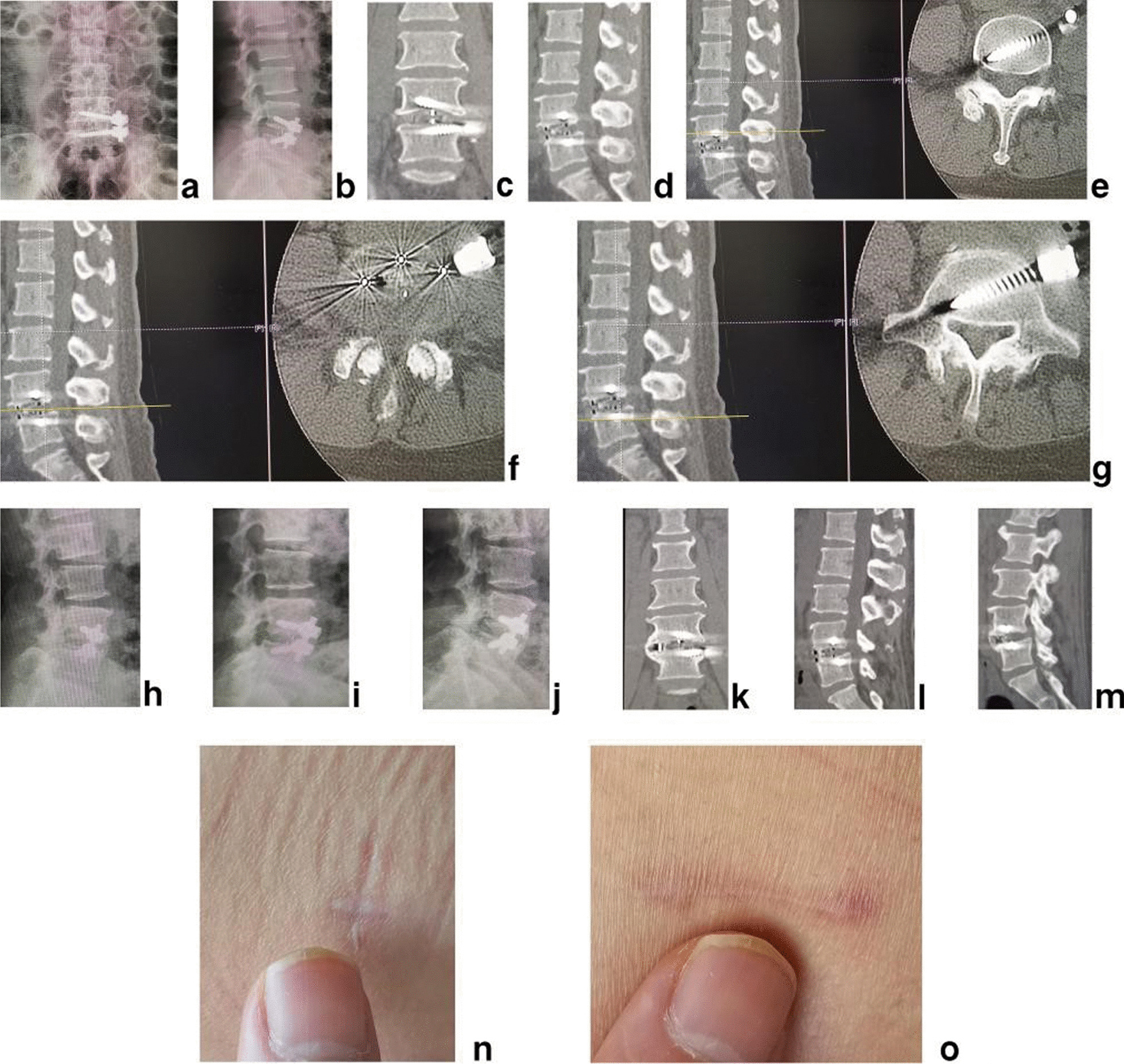
**a** Posteroanterior and **b** lateral X-ray image, **c** coronal, **d** sagittal and **e**–**g** axial CT image showed good postoperative position of cage and screws immediately after operation. On **h** lateral X-ray image there were no significant changes in intervertebral space height, lumbar lordotic angle and operative segmental lordotic angle 2 years after operation. No instability at fusion level was found on **i** hyperflexion and **j** hyperextension lateral X-ray image. Fusion grade at 2-year follow-up was grade I on **k** coronal and **l**, **m** sagittal CT images. The picture shows the cosmetic incisions for **n** PTES and **o** OLIF 2 years after operation

The postoperative radiographs and CT scans demonstrated good position of cage and screws (Fig. 5a–g). Postoperative intervertebral space height, lumbar lordotic angle and operative segmental lordotic angle were, respectively, 13.03 ± 1.54 mm(F = 7.634, *P* < 0.001),48.70 ± 8.37°(F = 38.082, *P* < 0.001) and 19.66 ± 3.04°(F = 95.869, *P* < 0.001). which were significantly more than those of preoperation. There were no significant changes in intervertebral space height, lumbar lordotic angle, operative segmental lordotic angle (Fig. 5h) and no instability at fusion level (Fig. 5i,j) 2 years after operation. Fusion grades based on the Bridwell grading system at 2-year follow-up were grade I (Fig. 5k-m) in 29 segments (76.3%), grade II in 9 segments (23.7%). No subsidence of cages and no failure of instruments were observed.

One patient encountered nerve root sleeves rupture during PTES and did not confront cerebrospinal fluid leakage or other abnormal clinical symptoms. There were two cases of hip flexion pain and weakness without anterior thigh numbness and other motor weakness, which was relieved during 1 week after surgery. No patients had any form of permanent iatrogenic nerve damage and a major complication.

## Discussion

Lin et al. [20] indicated in their study that OLIF can achieve indirect neurologic decompression by placing the big cage into disc space to increase disc height, which can tighten the posterior longitudinal ligament, enlarge the cross-sectional area (CSA) of spinal canal and intervertebral foramen and alleviate the pressure on neurologic elements. The radiographic study of Limthongkul et al. [21] showed that the CSA of thecal sac increased from 93.1 ± 43.0 mm to 127.3 ± 52.5 mm after OLIF. In the study of Beng et al. [22], the patients were divided into three groups based on their preoperative lumbar lordosis: group A, < 0°; group B, 0°-20°; and group C, > 20°. After OLIF, the mean CSA enlargement ratio was 27.5%, 32.1% and 60.4% in groups A, B and C, respectively. To some extent, these can relieve the patient’s symptoms in OLIF [23]. However, sometimes OLIF alone had no improvement of neurologic symptoms due to the inadequate decompression such as extreme severe lumbar canal stenosis and bony lateral recess stenosis [10, 12, 24–27]. When the segment is too rigid to be restored, it is difficult to obtain a greater postoperative disc height and more indirect decompression effect [28]. Additionally, if the free herniated disc migrates into spinal canal or head, or tail of the involved segment, or the culprit segment is not the instability one, OLIF has no efficacy of indirect decompression. Another entrance into operation room for neurologic decompression is needed, which maybe require another general anesthesia and increase the aggressiveness.

Most patients of multi-level LDDs with or without intervertebral instability have symptoms of single-level nerve root compression, such as unilateral or bilateral leg pain [16]. Sometimes there is intermittent claudication that unilateral or bilateral leg pain, numbness, discomfort or tiredness occurs after walking for 50 to 100 m, which could be relieved after a few minutes of rest. When lumbar disc herniation, lateral recess stenosis or intervertebral foramen, most patients have one leg pain and few have pain in different position of both legs coming from nerve root compression of different levels. When lumbar central spinal canal stenosis, there is bilateral pain in the symmetric position of both legs resulting from the compression of bilateral traversing nerve roots of same level. In 2017, we first introduced our PTES with reduced steps, simple orientation and easy puncture, which can significantly decrease the times of fluoroscopy projection and shorten the operation time [14]. We used PTES to successfully treat LDDs with neurologic symptoms including lumbar spondylolisthesis [14–16]. During the operation, we performed press-down enlargement of foramen to saw off the ventral bone of articular process. In addition, the hypertrophic ligamentum flavum and the protruding nucleus pulposus were removed to expand the lateral recess. Under endoscopy, the ipsilateral and contralateral traversing nerve roots could be exposed for central spinal canal decompression, and the bilateral nerve roots can be decompressed from one side through a small incision. In this study, we predicted the culprit segment according to the position of leg pain and performed PTES under local anesthesia to treat the culprit segment for direct decompression before OLIF. During PTES we used VAS to evaluate the relaxation sensation of involved leg after neurologic decompression. A VAS score of 0–3 means obvious relaxation and good efficacy, the treated segment is the culprit one, and the operation can be finished. A VAS score of 4–6 indicates moderate relaxation, the treatment is partially effective, and the treated segment is the culprit one, but other culprit segments need treatment. A VAS score of 7–10 shows mild or no relaxation and no efficacy, the treated segment is not the culprit one, and the culprit segment needs treatment until the VAS score decreases to 0–3 [16]. This method can guarantee surgical efficacy. The results showed PTES was performed for 1 segment in 31 cases including 25 cases of instability segment and 6 cases of no instability segment, for 2 segments including instability segment in other 7 cases. The leg pain VAS and ODI significantly dropped after surgery(*P* < 0.001), and there was no reoperation for neurologic decompression.

According to Soriano-Baron et al. [11], OLIF alone maintained axial compressive stiffness when comparing with the intact condition, and the surgeon should consider the biomechanical and patient-specific factors for selecting the appropriate supplement fixation technique for any interbody spacers. The study of Guo et al. [29] showed that OLIF alone could not provide sufficient stability and need additional fixation. Bilateral pedicle screw fixation(BPS) has the most rigid structure, but requires paraspinal muscle dissection and retraction during instrumentation, has neurologic risk, vascular injury, and increased operative time. Unilateral pedicle screw fixation (UPS) involve less damage to the paravertebral muscles, less perioperative bleeding and low instrument expense, but it offers significantly less stability than the BPS. Compared with BPS and UPS, lateral rod-screw fixation may be appropriate for patients with good bone quality, normal body mass index and nonspondylolisthetic lumbar fusion [29]. The additional posterior surgery of pedicle screws fixation enlarged the aggressiveness, prolonged the time of general anesthesia or furthermore required another general anesthesia. In this study, we inserted the pedicle screws into vertebral bodies and fixed the rod over screws from the anterolateral side after the placement of cage in the same mini-incision of OLIF. The results of our study showed that fusion grades based on the Bridwell grading system at 2-year follow-up were grade I in 29 segments (76.3%), grade II in 9 segments (23.7%), and no failure of instruments was observed. These confirmed that OLIF combined with anterolateral screws rod fixation can supply good biomechanical property for intervertebral fusion.

The cage used in OLIF is much bigger than that in PLIF or TLIF, which is beneficial for restoration of lumbar anatomy sequence. Postoperative intervertebral space height, lumbar lordotic angle and operative segmental lordotic angle were significantly more than those of preoperation (*P* < 0.001), and there were no significant changes 2 years after operation in this study. No subsidence of cage into vertebral body was found, which was related to protection of cortical endplate during preparation of intervertebral space. Attention must be paid to place the cage into disc space completely along with 3 directions of sagittal, axial and coronal plane of intervertebral space; otherwise, the tip of cage may be put into vertebral body through endplate and the subsidence of cage would happen.

**Fig. 6:**
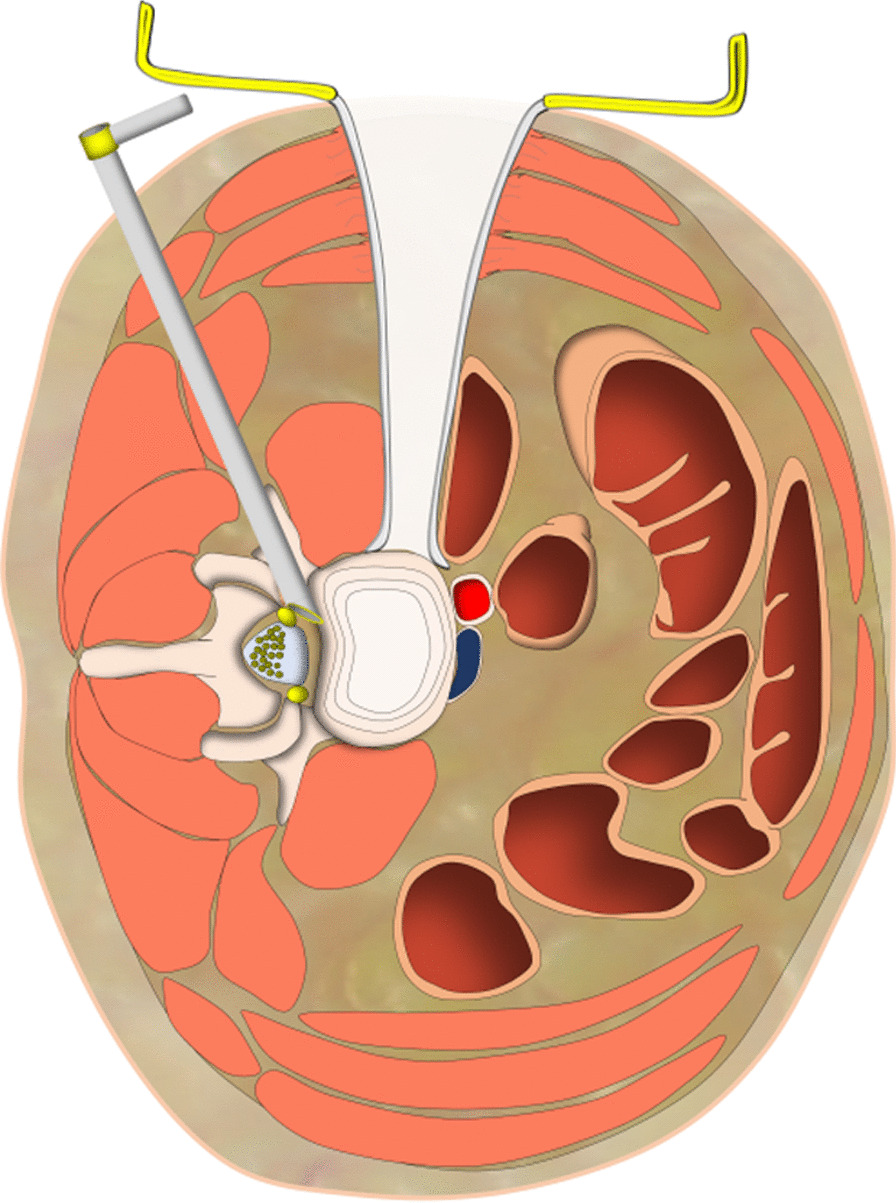
Lateral posterior approach of PTES and lateral anterior approach of OLIF meet with each other at posterior longitudinal ligament and annulus fibrosus of disc

We combined PTES under local anesthesia with OLIF and anterolateral screws rod fixation in the same mini-incision for the treatment of lumbar intervertebral instability. Posterolateral approach of PTES and anterolateral approach of OLIF meet with each other at posterior longitudinal ligament and annulus fibrosus of disc (Fig. 6). In PTES, the length of incision was 8.1 ± 1.1 mm (Fig. 5n) and only ventral bone of articular process was removed, which can be filled into the cage of OLIF. The natural corridor was utilized to place the cage, screws and rod through the incision of 40.0 ± 3.2 mm (Fig. 5o) for OLIF and anterolateral screws rod fixation. This hybrid surgery of two minimally invasive techniques can protect the paraspinal muscles and bone structures as much as possible, and there was only 30 (15–60) ml of blood loss. The frequency of intraoperative fluoroscopy during the operation was limited, and both the patients and surgeons were protected against the radiation injury. Compared with general anesthesia, local anesthesia has little influence on physical status. PTES was performed under local anesthesia, which did not prolong the time of general anesthesia for OLIF and anterolateral screws rod fixation. The operation duration under general anesthesia was only 69.2 ± 11.6 min. The natural corridor for OLIF and anterolateral screws rod fixation made postoperative drainage fluid little and when less than 20 ml/24 h the drain tube could be removed usually 1 or 2 days after the surgery. Patients could leave hospital as soon as possible, and the hospital stay was 4 (3–6) days.

Some authors reported the complications of lateral lumbar interbody fusion (LLIF) through transpsoas approach, such as anterior thigh pain, numbness, paresthesias or weakness due to direct muscle injury and injury to the lumbaplexus as it courses through the psoas [30]. OLIF through the approach between great vessel and psoas was introduced as an alternative procedure to the transpsoas approach, allowing for psoas preservation and avoids the lumbar plexus [7, 8]. In this study, two cases had hip flexion pain and weakness possibly related to traction of psoas muscles during OLIF and anterolateral screws rod fixation, which improved during 1 week after surgery. No symptoms of motor or sensory nerve injury such as anterior thigh numbness were observed, which indicates no lumbar plexopathies and nerve injury according to the study of sensory dermal zone by Ahmadian et al. [31]. Although there was nerve root sleeves rupture during PTES in one patient, no cerebrospinal fluid leakage or other abnormal clinical symptoms were found. Postoperative radiographs and CT scans showed that the position of cage and screws was good and no failure of instruments was observed during 2-year follow-up. No patients had any form of permanent iatrogenic nerve damage and a major complication. All these confirmed the safety of hybrid surgery.

## Conclusions

The hybrid surgery of PTES combined with OLIF and anterolateral screws rod fixation is a good choice of minimally invasive surgery for multi-level LDDs with intervertebral instability, which can get direct neurologic decompression, easy reduction, rigid fixation and solid fusion, and hardly destroy the paraspinal muscles and bone structures.

## Supplementary Information

**Additional file 1**. Case show: PTES combined with mini-incision OLIF and anterolateral screws rod fixation for the treatment of multi-level LDDs with L4 spondylolisthesis.
